# Transcriptional analysis reveals key insights into seasonal induced anthocyanin degradation and leaf color transition in purple tea (*Camellia sinensis* (L.) O. Kuntze)

**DOI:** 10.1038/s41598-020-80437-4

**Published:** 2021-01-13

**Authors:** Tony Kipkoech Maritim, Mamta Masand, Romit Seth, Ram Kumar Sharma

**Affiliations:** 1 Department of Biotechnology, CSIR-Institute of Himalayan Bioresource Technology, P.O. Box No. 6, Palampur, HP 176061 India; 2 Academy of Scientific and Innovative Research (AcSIR), CSIR-HRDC Campus, Ghaziabad, Uttar Pradesh 201 002 India; 3 Tea Breeding and Genetic Improvement Division, KALRO-Tea Research Institute, P.O. Box 820-20200, Kericho, Kenya

**Keywords:** Next-generation sequencing, Transcriptomics, Plant molecular biology, Secondary metabolism

## Abstract

Purple-tea, an anthocyanin rich cultivar has recently gained popularity due to its health benefits and captivating leaf appearance. However, the sustainability of purple pigmentation and anthocyanin content during production period is hampered by seasonal variation. To understand seasonal dependent anthocyanin pigmentation in purple tea, global transcriptional and anthocyanin profiling was carried out in tea shoots with two leaves and a bud harvested during in early (reddish purple: S1_RP), main (dark gray purple: S2_GP) and backend flush (moderately olive green: S3_G) seasons. Of the three seasons, maximum accumulation of total anthocyanin content was recorded in S2_GP, while least amount was recorded during S3_G. Reference based transcriptome assembly of 412 million quality reads resulted into 71,349 non-redundant transcripts with 6081 significant differentially expressed genes. Interestingly, key DEGs involved in anthocyanin biosynthesis [PAL, 4CL, F3H, DFR and UGT/UFGT], vacuolar trafficking [ABC, MATE and GST] transcriptional regulation [MYB, NAC, bHLH, WRKY and HMG] and Abscisic acid signaling pathway [PYL and PP2C] were significantly upregulated in S2_GP. Conversely, DEGs associated with anthocyanin degradation [Prx and lac], repressor TFs and key components of auxin and ethylene signaling pathways [ARF, AUX/IAA/SAUR, ETR, ERF, EBF1/2] exhibited significant upregulation in S3_G, correlating positively with reduced anthocyanin content and purple coloration. The present study for the first-time elucidated genome-wide transcriptional insights and hypothesized the involvement of anthocyanin biosynthesis activators/repressor and anthocyanin degrading genes via peroxidases and laccases during seasonal induced leaf color transition in purple tea. Futuristically, key candidate gene(s) identified here can be used for genetic engineering and molecular breeding of seasonal independent anthocyanin-rich tea cultivars.

## Introduction

Tea, (*Camellia sinensis* (L.) O. Kuntze), is an economically important plantation crop rich in flavor enhancing and health beneficial metabolites that also confer resistance to biotic and abiotic stresses^1^. Most of the elite tea cultivars grown worldwide are suitable for processing of the three popular types of commercial teas, viz*;* non-fermented (green tea), semi-fermented (oolong tea), and completely fermented (black tea)^2^. Global production of black and green tea is consistently increasing and projected to reach 4.42 and 3.31 million tons, respectively by 2027^3^. Despite the significant increase in global production, tea prices have stagnated or declined causing reduced profit margins and returns to the farmers^4^. Therefore, diversification of tea products can be an alternative way-out to raise demand and shoreup market prices. Nevertheless, diversification is greatly influenced by availability of suitable germplasm, wherein, anthocyanin-rich tea cultivars due to their pleasant taste and rich health beneficial compounds are getting global attention for production of “Specialty Tea” that can fetch premium prices to the farmers. Through breeding (natural or artificial hybridization) followed by clonal field selection, anthocyanin-rich tea cultivars (purple tea) have been developed for product diversification^5,6^. Anthocyanin, a water-soluble secondary metabolite synthesized in the cytosol and localized in vacuoles, is an important plant pigment responsible for the red, violet, purple and blue pigmentations in different plant tissues^7^. Their accumulations also play an important role in reducing damage caused by UV irradiation, photoinhibition and oxidative stress and more so enhance pollination^8^. Interestingly, various animal model studies have reported several health benefits including antioxidant, antiaging, anticancer and antidiabetic properties of anthocyanin-rich tea^9,10^. Therefore, anthocyanin-dependent purple coloration can be a potential morphological marker for breeding and selection of quality tea cultivars. Nevertheless, rapid degradation limits optimum utilization of anthocyanin-rich cultivars for production of specialty tea at commercial scale^11,12^. Therefore, understanding the molecular basis of anthocyanin degradation and leaf color transition is important for its genetic improvement. Previous metabolomic studies attributed the dynamic balance and ratio of different anthocyanin molecules, catechins and other plant pigments (chlorophyll and carotenoids) to variation in purple leaf coloration in tea^13,14^. Earlier molecular studies have also identified key genes/proteins and various transcription factors involved in anthocyanin biosynthesis in tea^15,16^. Likewise, few previous studies focused on developmental transition associated flavonoid/anthocyanin accumulation and leaf color change to attainment of maturity^17–20^. Additionally, recent studies in various plant species have revealed the involvement anthocyanin degradation enzymes [beta-glucosidases, polyphenol oxidase (PPO), class III peroxidases (POD) and laccases], and well-coordinated plant hormone signaling pathways in regulation of anthocyanin accumulation^21,22^. Interestingly, the role of PPO, POD and beta-glucosidases in oxidation of flavonol glycosides responsible for bitter-taste and astringency in tea was recently reported^23^. However, their role in anthocyanin accumulation in tea is not known. Moreover, the mechanism controlling seasonal induced anthocyanin degradation and purple leaf color transition remains elusive in tea. Therefore, transcriptome profiling of anthocyanin-rich tea during different seasons can provide deeper understanding of the molecular mechanism controlling seasonal induced leaf color transition in purple tea.

Annual tea production in India is broadly categorized into early flush (April to mid-June: S1), main flush (mid-June to mid-September: S2) and backend flush (mid-September to October: S3) seasons with availability of the best quality tea during S1^24^. Despite recording better yields, the main flush (S2) is compromised in terms of quality. Therefore, development and commercialization of “anthocyanin-rich tea” can be the potential alternative for increasing profitability of the tea sector. Interestingly, long-term field evaluation of anthocyanin-rich tea cultivars reveals purple leaf color transitions from reddish purple (RP) during S1 to dark gray purple (GP) during S2 and, subsequently turn moderately olive green (G) during S3.

In the present study, seasonal dependent total anthocyanin accumulation (TAC) vis-à-vis global transcriptional analysis of anthocyanin-rich tea during early (S1_RP), main (S2_GP) and backend (S3_G) flush seasons was performed to elucidate the dynamics of leaf color transitions in pigmented tea. Global gene expression analysis was performed for successful identification of key structural and regulatory genes involved in seasonal-induced biosynthesis/degradation of anthocyanin in purple tea. To the best of our knowledge, this is the first study to report on comprehensive elucidation of seasonal induced anthocyanin degradation, phytohormonal regulation and role of repressor TFs in anthocyanin accumulation in tea. This dataset forms a platform for advance understanding of molecular regulation of anthocyanin accumulation in different seasons and can be used futuristically for molecular breeding of seasonal independent anthocyanin-rich tea cultivars.

## Results

### Differential accumulation of total anthocyanin content (TAC)

**Figure 1:**
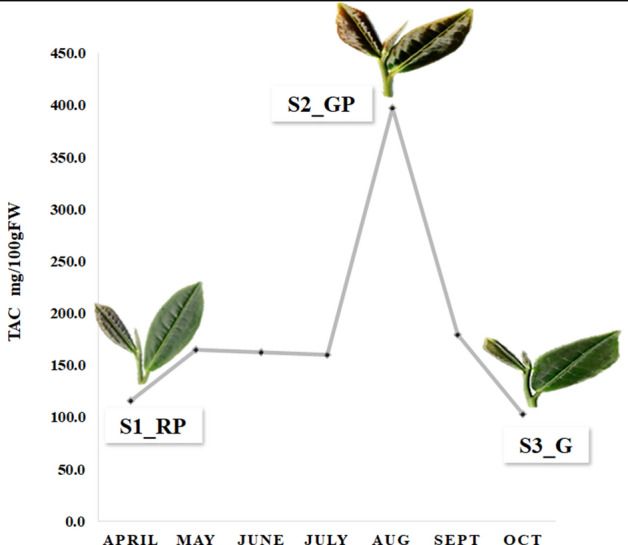
Leaf color transition and variation in total anthocyanin content.

To determine the variation in total anthocyanin dependent leaf color transitions in tea, spectrophotometric analysis of TAC was performed on samples collected monthly from April to October. Analysis of tea shoots with two leaves and a bud recorded clear difference in leaf color from reddish purple (RP) during April (S1_RP) to dark-gray purple (GP) in August (S2_GP) and moderately olive green (G) in October (S3_G)]. Similarly, TAC recorded significant increase from 115.0 mg 100 g^−1^ FW in April (S1_RP) to 397.0 mg 100 g^−1^ FW in August (S2_GP) before declining to 103.0 mg 100 g^−1^ FW in October (S3_G) (Fig. 1).

### Transcriptome sequencing

Transcriptome sequencing of three cDNA libraries representing reddish purple (S1_RP), dark-gray purple (S2_GP) and moderately olive green (S3_G) leaf coloration yielded 412 million high quality reads with ~ 95% quality score. Reference based mapping of quality reads resulted into 88.4% (S1_RP), 87.5% (S2_GP) and 86.4% (S3_G) of sample-wise reads that were successfully mapped to *Camellia sinensis* var. *sinensis* (CSS) genome^25^. Overall, 71,349 non-redundant transcripts with an average length of 1514.14 bp were identified in reference-based transcriptome assembly to the CSS^25^. Other assembly attributes such as N50 (1965 bp) and GC content (42.99%) provided high quality and quantity assurance of current RNA-seq data for downstream analysis (Supplementary Table S1).

### Functional annotation

**Figure 2:**
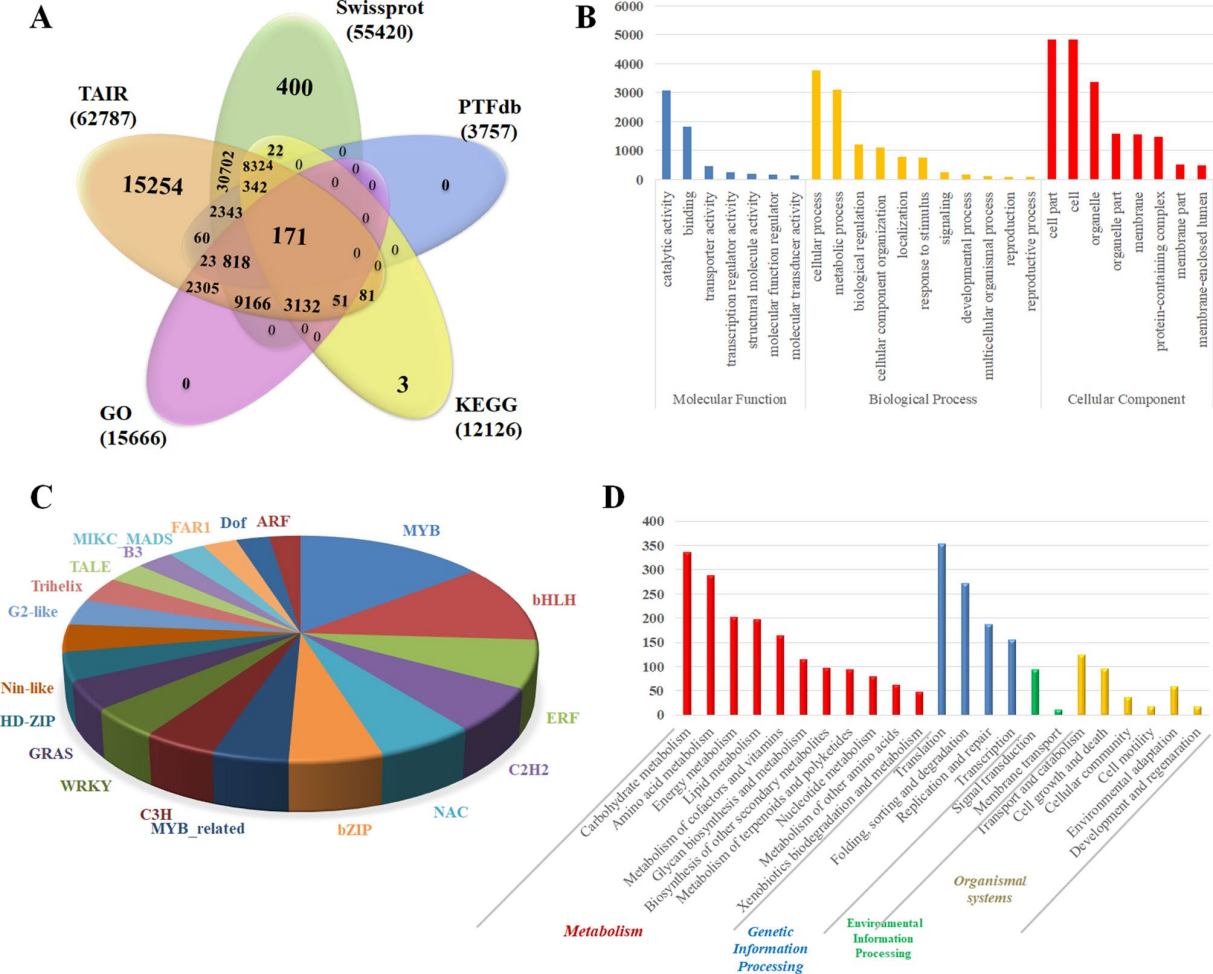
(**A**) Venn diagram of annotated transcripts in multiple databases. (**B**) Gene ontology classification of transcripts. (**C**) Frequency distribution of transcription factors in transcriptome data. (**D**) Functional classification and pathway assignment of assembled transcripts by KEGG.

To determine the putative functions of assembled transcripts, similarity search with mutiple public databases using BLASTX algorithm (e-value cut-off ≤ 1e−5) annotated 62,788 (87%), 55,420 (77.7%), 12,126 (12%), transcripts in TAIR, SwissProt, and KEGG respectively (Fig. 2a). Gene ontology (GO) terms were assigned to 40,935 (57.4%) transcripts, classified into biological process (13,203; 32.2%), molecular function (13,019; 31.8%) and cellular component (14,713; 35.9%) (Fig. 2b). Furthermore, annotation with plant-TFdb identified 3757 transcripts factors representing 56 TF families with abundance of MYB (430), basic-helix–loop–helix class (330) and Ethylene response factors (ERF) (201) (Fig. 2c). NR transcripts successfully assigned to KEGG pathways were also classified into five functional groups with abundance of pathways involved in plant metabolism (Fig. 2d).

### Differential gene expression analysis

**Figure 3:**
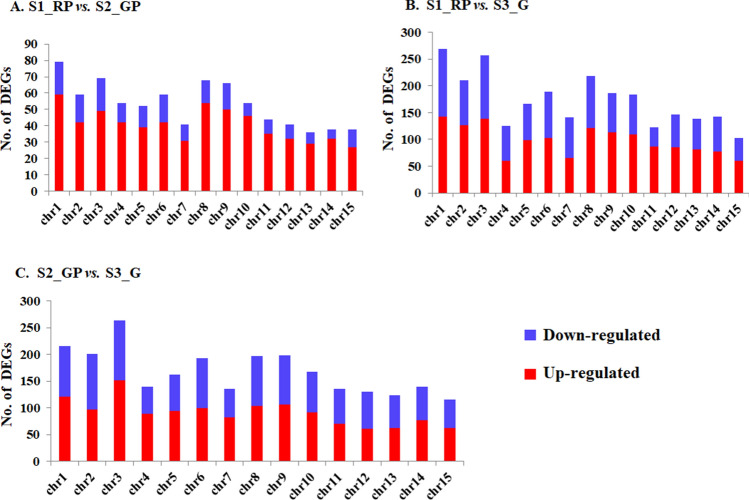
Differentially expressed genes identified by pairwise comparison and their chromosomal distribution in the CSS genome. Red and blue bars represent up-regulated and down-regulated DEGs, respectively.

To unravel global insight of differentially expressed genes during leaf color transitions, pairwise comparisons between S1_RP, S2_GP and S3_G identified ~ 6081 DEGs, assigned to different chromosomes (*Chr*) of the CSS genome^26^ (Fig. 3). 818 DEGs including 626 upregulated and 192 downregulated identified in S1_RP vs*.* S2_GP comparison were mostly mapped to *Chr*1 (79 DETs), *Chr*3 (69 DEGs), *Chr*8 (68 DEGs) and 38 DEGs each to *Chr*14 and *Chr*15. Similarly, S1_RP vs*.* S3_G comparison revealed 2668 DEGs (1506 upregulated and 1162 downregulated) mainly assigned to *Chr*1 (269 DEGs), *Chr*3 (257 DEGs), and least in *Chr*11 (122 DEGs) and *Chr*15 (103 DEGs). Likewise, 2595 DEGs (1421 upregulated and 1174 downregulated) identified in S2_GP vs*.* S3_G comparison recorded maximal mapping to *Chr*3 (263 DEGs), and least number of DEGs (115) to *Chr*15.

### GO and KEGG enrichment analysis

GO enrichment analysis using Tea Plant Information Archive (http://tpia.teaplant.org/) identified 136 significantly (FDR ≤ 0.05) enriched GO terms in S1_RP vs*.* S2_GP (50), S1_RP vs*.* S3_G (36) and S2_GP vs*.* S3_G (50) comparisons (Supplementary Table S2). In the biological process category, unique enrichment of ‘phenylpropanoid metabolic process’, ‘secondary metabolite biosynthesis’ ‘reactive oxygen species metabolic process’ and ‘lignin biosynthetic process’ in S1_RP vs. S2_GP comparison suggest their role in anthocyanin biosynthesis. While, significant enrichment of ‘oxidation–reduction process’, ‘response to oxidative stress’ and ‘circadian rhythm’ in S2_GP vs*.* S3_G suggest possible role in anthocyanin degradation. Likewise, in the molecular function category ‘binding’, ‘peroxidase activity’ and ‘antioxidant activity’ recorded optimum enrichment during reddish purple to dark grey purple leaf color transitions (S1_RG vs*.* S2_GP), whereas, enrichment of ‘oxidoreductase activity’, ‘secondary active transmembrane transporter activity’ and ‘microtubule binding’ in S2_GP vs*.* S3_G, suggesting their possible role in anthocyanin degradation. Similarly, cellular component associated with cell wall and membrane including ‘Apoplast’ were enriched significantly in S1_RG vs. S2_GP, while enrichment of ‘chloroplast thylakoid membrane’, ‘photosynthetic membrane’ and ‘plastid thylakoid membrane’ in S2_GP vs. S3_G suggests the critical role of cell wall and membrane organelles in regulating anthocyanin accumulation in tea. Additionally, ‘glucan metabolic process’, ‘aminoglycan metabolic process’ and ‘cell wall macromolecule metabolic process’ (biological processes), ‘catalytic’, ‘hydrolase’, ‘transferase’ and ‘oxidoreductase’ activities (molecular functions) and external encapsulating structure’, ‘cell wall’ and ‘extracellular region’ (cellular component) were significantly enriched irrespective of the pairwise comparisons.

Furthermore, KEGG enrichment analysis identified 55 metabolic pathways including 11 in S1_RP vs. S2_GP; 27 in S1_RP vs. S3_G and 17 in S2_GP vs. S3_G, (Supplementary Table S3). Metabolic pathways, such as ‘cyanoamino acid metabolism’, ‘diterpenoid biosynthesis’, ‘N-glycan biosynthesis’ and ‘oxidative phosphorylation’, ‘photosynthesis’ and ‘pyruvate metabolism’ were significantly enriched in S1_RP vs. S3_G, and S2_GP vs. S3_G suggesting potential role in anthocyanin degradation, while, ‘isoflavonoid biosynthesis’ uniquely enriched in S1_RP vs. S2_GP may be linked to anthocyanin biosynthesis. Interestingly, important metabolic pathways like ‘plant hormone signal transduction’, ‘phenylpropanoid biosynthesis’, ‘starch and sucrose metabolism’, ‘flavonoid biosynthesis’, ‘MAPK signaling’ and ‘Sesquiterpenoid and triterpenoid biosynthesis’ also recorded significant enrichment irrespective of the pair-wise comparisons. Among these, key pathways such as ‘plant hormone signal transduction’, ‘phenylpropanoid biosynthesis’ and ‘flavonoid biosynthesis’ were considered for downstream analysis to elucidate anthocyanin biosynthesis and degradation in tea.

### Gene expression analysis of candidates related to anthocyanin biosynthesis

**Figure 4:**
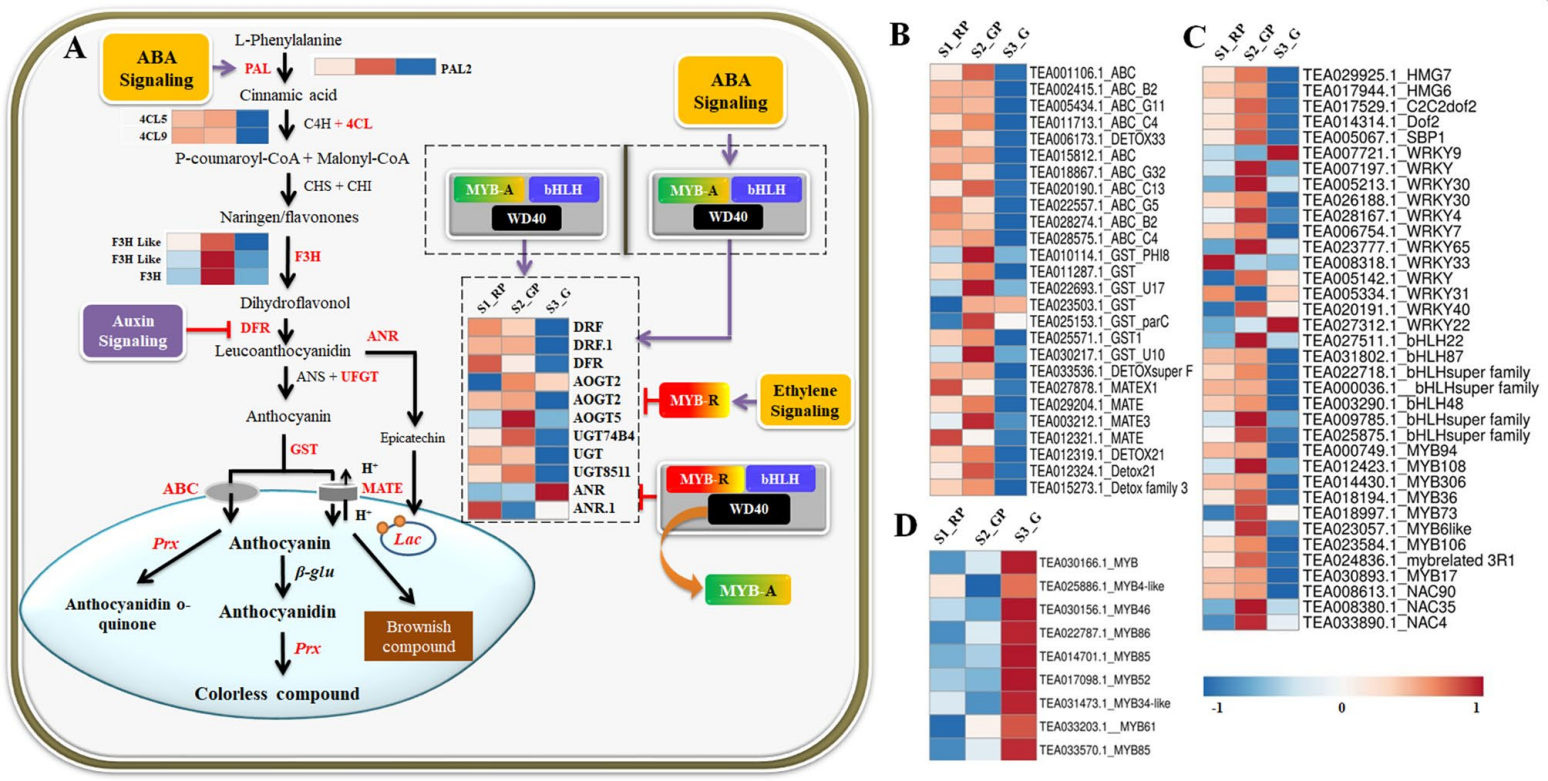
(**A**) Anthocyanin biosynthesis pathway. Highlighted genes (red) represent differentially expressed genes of the pathway. Purple pointed arrows represent activation, curved right arrow represent displacement while red bar-headed arrows represent inhibition. (**B**) Heatmap of differentially expressed transporters. (**C**) Transcription factors associated with anthocyanin biosynthesis in tea. (**D**) Putative repressor MYBs. Blue and red colors of heatmaps represent upregulated and downregulated expression levels, respectively. The color scale reflects log2 transformed FPKM values.

**Figure 5:**
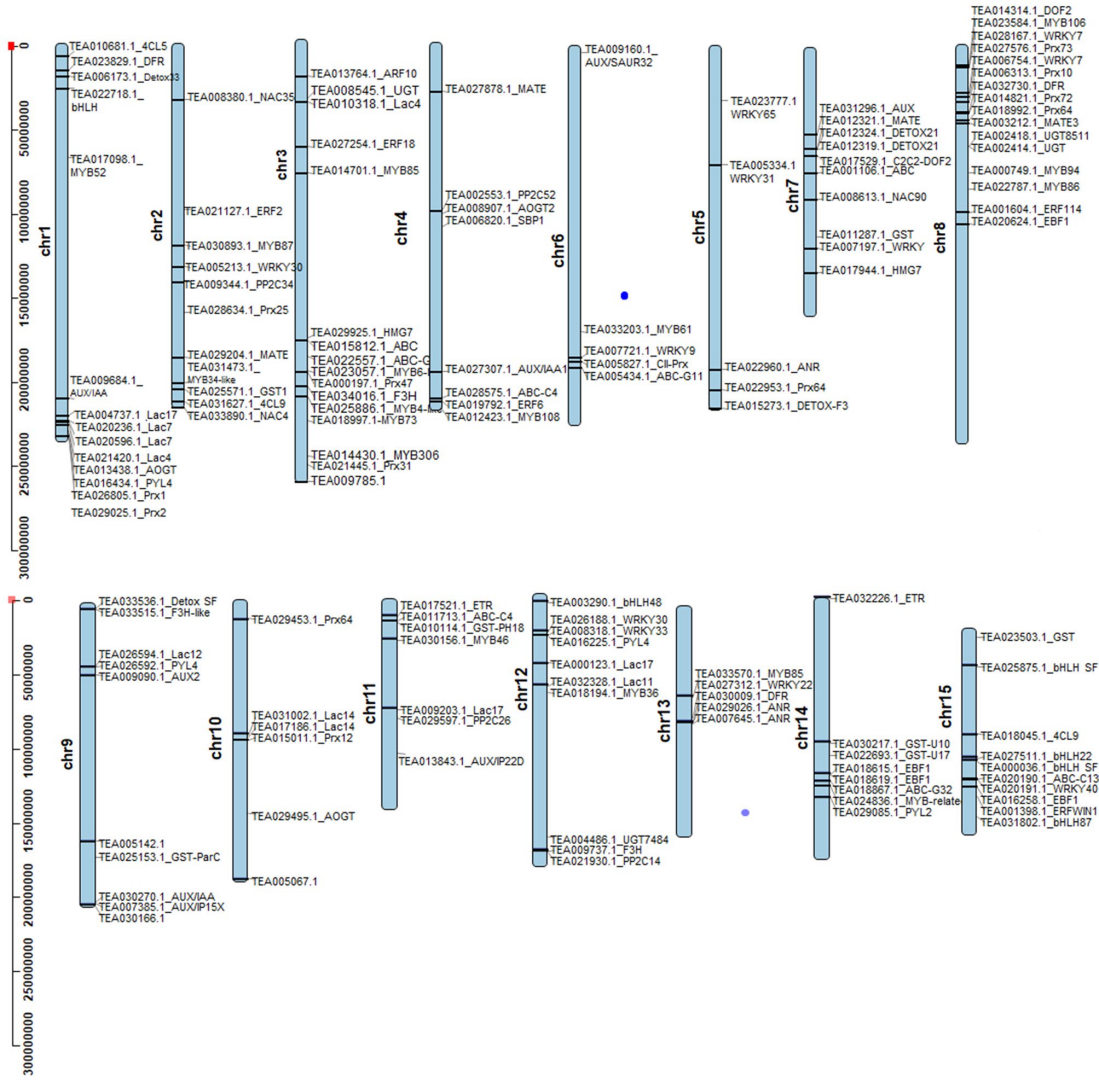
Chromosomal distribution of DEGs involved in anthocyanin biosynthesis and degradation in tea.

Considering key role of phenylpropanoid/flavonoid pathway in anthocyanin biosynthesis, 20 transcripts encoding early biosynthetic genes (EBGs); phenylalanine ammonia-lyase (PAL)*,* 4-coumarate-CoA ligase (4CL)*,* flavanone 3-hydroxylase (F3H) and late-biosynthetic genes (LBGs); dihydroflavonol 4-reductase(DFR)*,* anthocyanidin reductase (ANR) and anthocyanin 3′-*O*-beta-glucosyltransferase (AOGT), UDP-glycosyltransferase (UGT/UFGT*)* of the anthocyanin biosynthesis were differentially expressed (Supplementary Table S4; Fig. 4A). Their upregulation in S2_GP and downregulation in S1_RP and S3_G, was strongly correlated to TAC (Supplementary Table S5). Interestingly, upregulated expression of anthocyanin reductase (*ANR*) reportedly involved in epicatechin biosynthesis^27^ was negatively correlated with TAC during S3_G indicating their important role in regulating metabolic flux towards anthocyanin biosynthesis in tea. Moreover, distribution of anthocyanin biosynthesis genes in 11 of 15 chromosomes (Fig. 5) including *Chr*1,* Chr*2,* Chr*3 and *Chr*10 successfully mapped with quality related QTLs, suggests close linkage between anthocyanin and other quality related parameters in tea^26^.

Furthermore, differential expression of 26 candidate genes involved in anthocyanin transport (Supplementary Table S4) including 10 ATP-binding cassette transporters (ABC)*,* 9 multidrug and toxic extrusion transporter (MATE) and 7 glutathione *S*-transferase (GST), mostly upregulated in S2_GP, suggests the critical role of transporters in regulation of anthocyanin biosynthesis (Fig. 4B). Interestingly, strong positive correlation between upregulated expression of key transporters and TAC (Supplementary Table S5) suggest transporter mediated accumulation of anthocyanin in tea. Most of these transporters were assigned to *Chr*7 and *Chr*11 of CSS genome^25^.

### Dynamic expression of regulatory genes

Anthocyanin biosynthesis is greatly influenced by dynamic expression of transcription factors (TFs) and phytohormones.

### Transcription factors (TFs) mediated regulation of anthocyanin

Transcription factors (TFs) are master-proteins controlling key biological processes such as, metabolism, growth, biotic and abiotic stress responses^28^. In this study, 46 differentially expressed (DE) TFs including MYB and MYB-related (18), WRKY (12) and bHLH (7), NAC (3), C2H2 (2), HMG (2) and SBP (2) were identified during leaf color transitions of purple tea (S1_RP, S2_GP, S3_G) (Supplementary Table S6; Fig. 4C). TFs including MYB (MYB306), bHLH (bHLH3, bHLH48, bHLH87 and bHLH94) exhibited strong positive correlation (> 0.99) with key genes of anthocyanin biosynthesis (4CL5, ANR and AOGT) and TAC (Supplementary Table S5). Furthermore, strong positive correlation (> 0.9) between HMG TFs (HMG-B7, B8) with EBGs (PAL2, 4CL5) and LBGs (UGT7AB4), UGT8511) suggests their key role in anthocyanin biosynthesis (Supplementary Table S5). While, strong negative correlation between expression pattern (Fig. 4D) of MYBs (MYB4-like, MYB85, MYB86) and genes of anthocyanin biosynthesis pathway (PAL2, UGT7AB4, UGT8511) and TAC suggest key role of MYB TFs as “transcriptional repressors”. Furthermore, successful assignment of key DE TFs (bHLH, MYB87, MYB73) to *Chr*1, *Chr*2, and *Chr*3 (Fig. 5) harboring known quality related QTLs further affirmed their regulatory role in anthocyanin biosynthesis in tea. Interestingly, current study, for first time reveals possible role of HMG, NAC, C2H2 and SBP TFs in regulation of anthocyanin biosynthesis in tea.

### Phyto-hormonal regulation of anthocyanin biosynthesis

**Figure 6:**
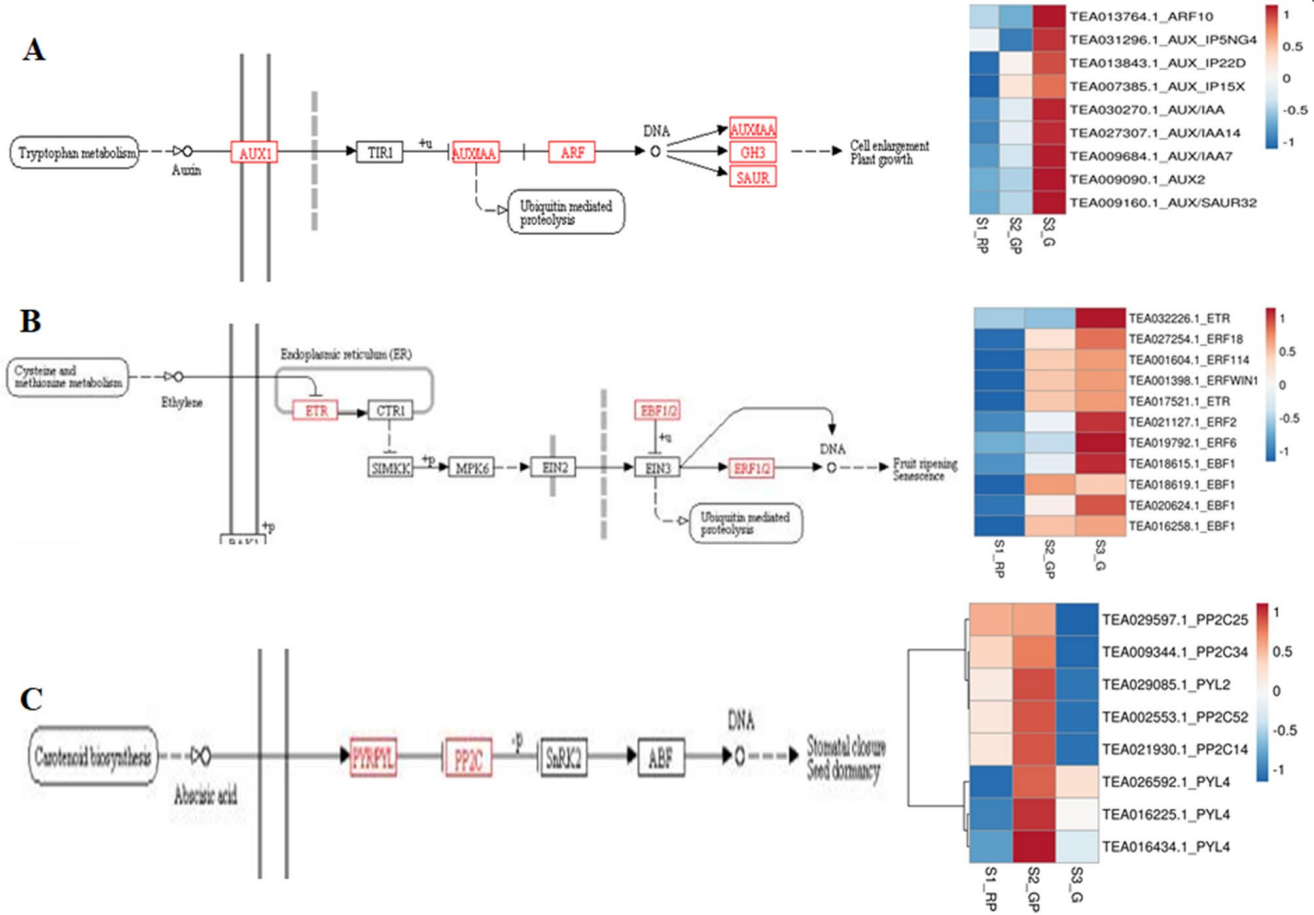
Differential expression of key genes involved in phytohormone signaling during leaf color transition. (**A**) Auxin acid signaling genes. (**B**) Ethylene signaling genes. (**C**) Abscisic acid signaling genes. Phytohormone signaling pathways were obtained from KEGG^61^ (https://www.kegg.jp/kegg/kegg1.htm).

Abscisic acid (ABA), auxin and ethylene signaling transduction pathways are key regulators of anthocyanin biosynthesis^22^. In this study, 28 DE genes of plant hormone signal pathways including those involved in auxin (9), ethylene (11) and Abscisic acid (8) signaling were identified (Supplementary Table S4). Auxin signaling regulators including 4 auxin responsive proteins (AUX/IAA/SAUR), 3 auxin induced proteins, and one each of auxin response factor (ARF) and auxin transporter-like protein spanning to *Chr*9,* Chr*7,* Chr*6,* Chr*11,* Chr*3,* Chr*1 and* Chr*4 were upregulated during S3_G and downregulated in S2_GP (Fig. 6A). Moreover, ethylene signaling pathway genes such as ethylene receptor (ERS: 2), ethylene response factor (ERF; 5) and EIN3-binding F-box protein (EBF1/2; 4) exhibited upregulated expression in S3_G, while downregulated in S2_GP and S1_RP (Fig. 6B). Furthermore, expression pattern of EBF and ERS regulators mapped to *Chr*14,* Chr*15,* Chr*3,* Chr*2 *Chr*11,* Chr*8 and *Chr*4 exhibited strong negative correlation (> 0.999) with TAC content (EBF1c and ERS1b), photoperiod (EBF1a), relative humidity (EBF1d) and precipitation (EBF1c and ERS1b) suggesting inhibitory role of ethylene and auxin in light-induced anthocyanin accumulation in tea (Supplementary Table S5). Contrarily, genes involved in ABA signaling [abscisic acid receptor (PYL4) and protein phosphatases (PPP2C_34)] recorded upregulated and downregulated expressions during S2-GP and S3_G, respectively (Fig. 6C). Strong positive correlation (> 0.99) with TAC, relative humidity (PYL4) and photoperiod (PP2C_34) indicated their important role in ABA assisted anthocyanin accumulation during seasonal leaf color transitions in tea^29^.

### Expression analysis of candidate genes involved in anthocyanin degradation

**Figure 7:**
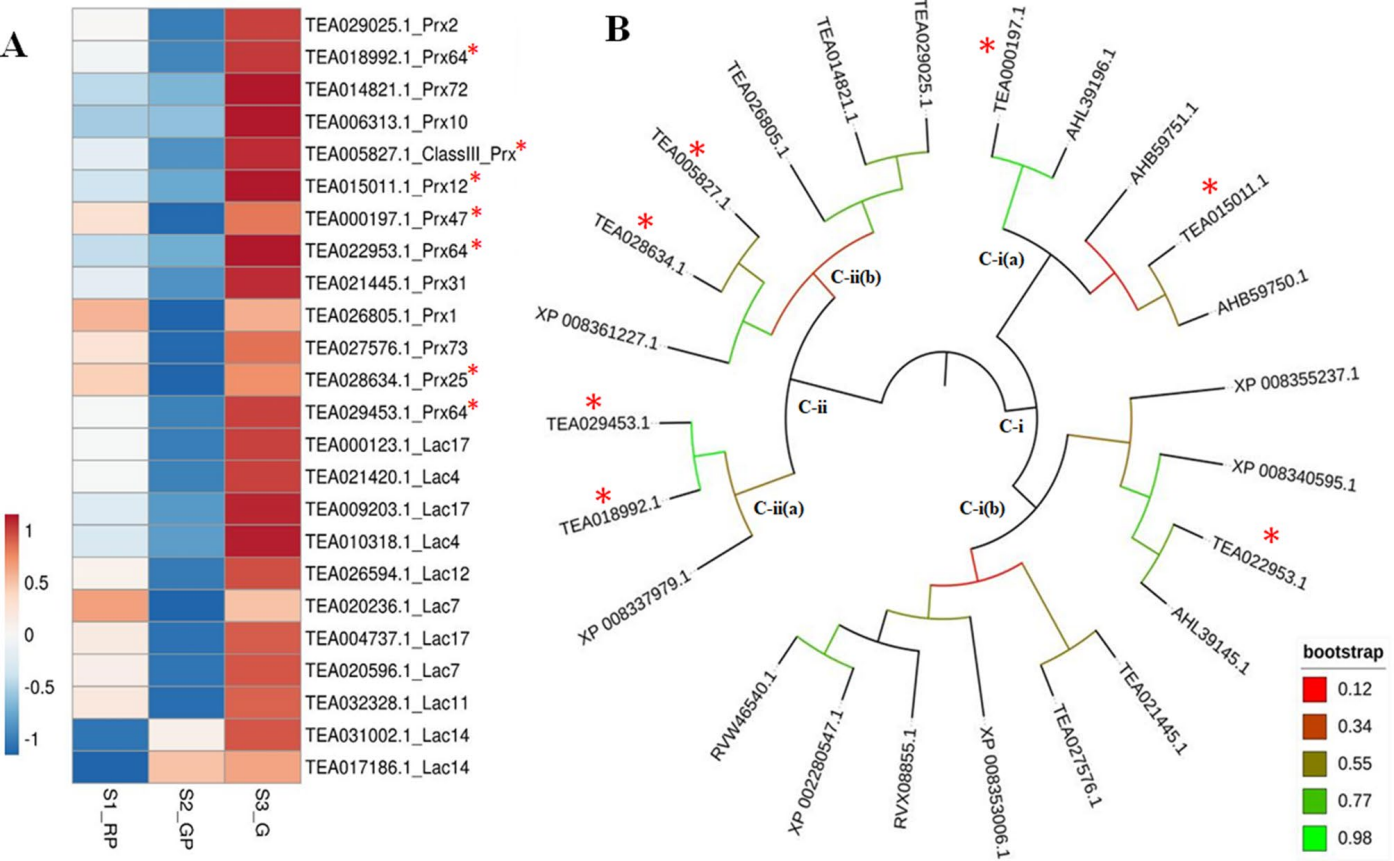
(**A**) The expression pattern of DE candidate peroxidases and laccases. (**B**) Phylogenetic tree of DE putative peroxidases and functionally characterized peroxidases. The * indicates tea peroxidases exhibiting significant homology with known peroxidases.

Intensity of purple leaf coloration is dependent on the net results of biosynthesis and degradation of anthocyanin. In this study, 24 DE key laccases (*Lac*; 11) and peroxidases (*Prx;* 13) involved in anthocyanin degradation were identified and mapped to *Chr*1 and *Chr*8 (Supplementary Table S4). The expression pattern of candidate anthocyanin degrading enzymes exhibited significant upregulation during S3_G as compared to S2_GP and S1_RP (Fig. 7A). Nevertheless, their optimum expression in S3_G along with negative correlation (0.769) with total anthocyanin content suggests their involvement in anthocyanin degradation and eventual loss of purple colour in tea cultivars (Supplementary Table S5). Moreover, strong negative correlation (> 0.99) identified between the expression of laccases (*lac*12, *lac*17 and *lac*4) and peroxidases (*Prx*31 and *Prx*64) with photoperiod also suggests light dependent anthocyanin degradation in tea. Furthermore, successful assignment of laccases and peroxidases preferably towards the ends of the chromosomes suggest their significant involvement in influencing genetic variation of desirable traits including leaf color transition in tea (Fig. 5).

### Phylogenetic analysis of differentially expressed peroxidases

Phylogenetic analysis of 13 DE key putative peroxidases involved in anthocyanin degradation with corresponding functionally characterized peroxidases of Populus, Apple and Brunfelsia clustered into two main clades (I and II) each with sub-clades (a and b) (Fig. 7B). In sub-clade I(a), TEA000197.1 recorded significantly higher homology with known anthocyanin degrading class III peroxidase in populous [AHL39196.1], while TEA015011.1 was clustered with Brunfelsia peroxidase [AHB59750.1 BcPrx02]. Furthermore, significantly higher homology was revealed between tea peroxidase [TEA022953.1] and class III peroxidase of populous [AHL39145.1] as grouped together in sub-clade I(b). Similarly, clade II revealed significant homology between tea peroxidases [TEA029453.1 and TEA018992.1] and apple (*Malus domestica*) peroxidase 64 [XP008337979.1] in sub-clade II (a)*,* whereas, tea peroxidases [TEA028634.1 and TEA0005827.1] and apple (*Malus domestica)* peroxidase 25 [XP008361227.1] were grouped together in sub-clade II(b). Significant homology between candidate peroxidases with known anthocyanin degrading peroxidases (CIII Prx, BcPrx02, AHL39196.1, AHB59750.1) speculate key role of peroxidases in anthocyanin degradation in tea^30^.

### qRT-PCR validation of DEGs

**Figure 8:**
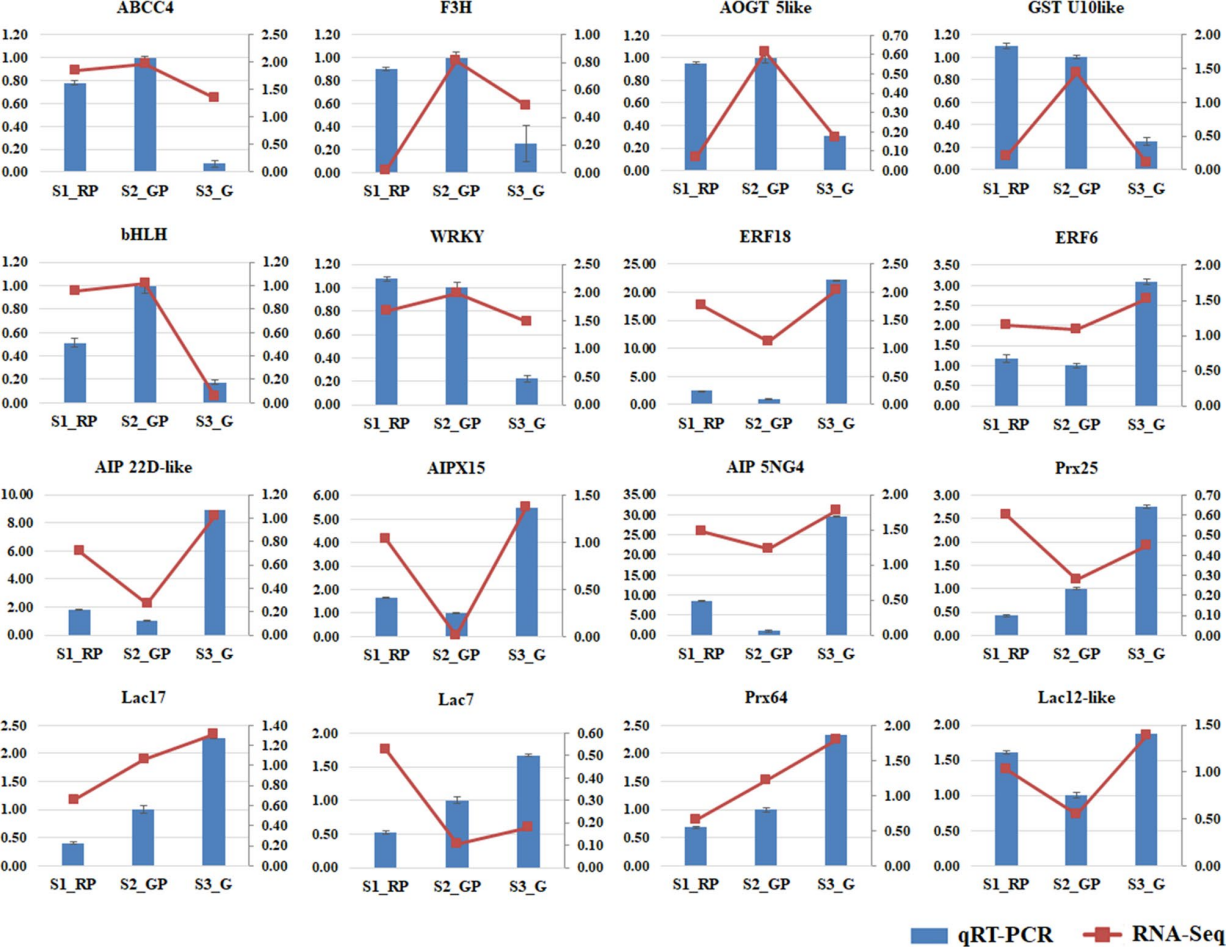
The expression profiles of 16 DEGs as determined by qRT-PCR and their correlation with RNA-seq expression data.

To confirm expression patterns of candidate genes identified in transcriptome dataset, sixteen key DEGs (Supplementary Table S7) involved in major pathways of anthocyanin accumulation were successfully validated in qRT-PCR analysis. The expression trends corresponded to RNA-seq expressions confirming the reliability of current RNA-seq data and computational analysis (Fig. 8). Interestingly, qRT expression patterns of validated genes were also consistent with TAC dynamics during leaf color transitions in tea (S1_RP, S2_GP, S3_G).

## Discussion

The dynamics of biosynthesis and degradation of anthocyanin together may influence changes in composition and ratio of different anthocyanin molecules during leaf color transitions^31^. In this study, strong positive correlation between leaf color changes [reddish purple (S1_RP), dark gray purple (S2_GP) and moderate olive green (S3_G)] and TAC, clearly distinguished dynamics of seasonal induced anthocyanin accumulation as recently reported in tea and, raspberry^6,32^. Optimum accumulation of anthocyanin during S2_GP can be explored as “potential season” for production of “specialty anthocyanidin-rich green tea” that fetches premium price at commercial scale^6^. Moreover, key genes identified in this study can be validated and utilized for genetic improvement of TAC in tea plants during seasons of low synthesis. Therefore, genome-wide elucidation of molecular mechanism of seasonal induced leaf color transitions can be key to sustain anthocyanin accumulation and prevent degradation for seasonal independent commercial production of “specialty tea”. High throughput deep transcriptome sequencing generated ~ 412 million high quality/clean reads assembled into 71,349 transcripts with homology based functional annotation > 85% in multifarious public databases, indicating improved annotations and accuracy of the assembly during this study^33^. Furthermore, significant enrichment of biological functions (secondary metabolite biosynthesis, oxidoreductase activity) and pathways related to anthocyanin biosynthesis and degradation; ‘plant hormone signal transduction’ and ‘phenylpropanoid/flavonoid biosynthesis’ suggests their active contribution in anthocyanin accumulation and seasonal dependent leaf color transition in tea. Additionally, significant positive correlation between qRT-PCR and RNA-seq expression data of 16 DE candidate genes confirm the reliability and utility of current RNA-seq data for genomic studies in tea.

Upregulated expression of PAL, 4CL, F3H, AOGT and UFGT during S2_GP affirmed their critical role in anthocyanin biosynthesis^34^. Similarly, significant positive correlation between AOGT5, F3H_Like, PAL2 genes and TAC suggest efficient performance of the pathway leading to enhanced anthocyanin accumulation during S2_GP. Interestingly, current findings are in concordance with recent report on seasonal dependent responses of anthocyanin-rich tea cultivars in tea^6^. Additionally, dynamic expression of AOGT and UFGT, upregulation during S2_GP, and downregulation during S3_G, suggest their role as key regulators enhancing the hydrophilicity and stability of vacuolar accumulation of anthocyanin in tea^35^. Similarly, upregulated expression of ANRs, catalyzing the branching step leading to biosynthesis of epicatechin, during S3_G suggest possible involvement in negative regulation of anthocyanin pathway due to limited/non-availability of substrate flux, (leucoanthocyanidin) for anthocyanin biosynthesis^27^. Moreover, upregulated expression of ANR during S3_G may be associated with increasing substrate availability for laccase-mediated anthocyanin degradation in tea. Additionally, glutathione *S*-transferase (GST), ABC and MATE play key role in anthocyanin transportation from the cytosolic face of endoplasmic reticulum (biosynthesis site) through a glutathione *S*-transferase (GST) mediated transport mechanism in which anthocyanin is conjugated with glutathione by GST and moved to vacuolar surface, where ABC and MATE transporter proteins transfer them to the vacuole (storage site)^36^. ABC transporters are reportedly involved in ATP dependent transportation of glucosylated anthocyanidin (malvidin-3-*O*-glucoside), while MATE mediate transport of manonylated anthocyanidins in the presence of electrochemical gradients of H^+^ or other ions created by V-ATPase or V-PPase^37,38^. Furthermore, ABC and MATE are considered primary and secondary active transport systems in plants, respectively^39^. In this study, upregulated expression of key transporters during purple leaf colour transitions of S1_RP and S2-GP and down regulated expression during S3_G (moderately olive green) suggests well-coordinated seasonal dependent GST mediated anthocyanin accumulation in tea. Interestingly, functional validation GST in a mutant *Vitis vinifera* line (defective of GST) confirmed its key role in anthocyanin accumulation^40^. Additionally, significantly higher expression of ABC transporters during S2_GP corroborates previous reports on their important role in anthocyanin accumulation in purple leaf tea cultivars^41^.

Furthermore, expression of important TFs including MYBs (18) and bHLH (7) may be associated with activation or repression of EBGs (MYBs) and LBGs (MYB-bHLH-WD40) of anthocyanin pathway^42^. Previous studies indicate that R2R3-MYB (MYB11, MYB12 and MYB111) are directly involved in the activation of EBGs (CHI, CHS, F3H and F3′H), while MBW (MYB-bHLH-WD40) complex activates LBGs such as DFR, ANS and UFGT^43^. Therefore, strong positive correlation between expression of key TFs (MYB and bHLH), anthocyanin biosynthetic genes and TAC during S2_GP reported here, are in accordance to earlier studies in tea^44^.

Considering key role of MBW complex and WRKY in vacuolar regulation of pH through controlled expression of proton pumps, upregulated expression of MYBs, bHLH and WRKY TFs may be associated with increased acidity in the vacuole enhancing purple colour pigmentation during S2_GP^7^. Nevertheless, their downregulation possibly reduced acidity of the vacuole resulting in reduced intensity of anthocyanin purple coloration during S3_G. Moreover, upregulation of novel NAC95, NAC4, NAC35, HMG6 & 7, DOF2 and SBP1 TFs during S2_GP may be associated with transcriptional activation of gene cascades involved in anthocyanin biosynthesis in tea^16^.

MYB TFs are also involved in transcriptional repression in the biosynthesis of phenylpropanoid-derived compounds with ~ 37 repressor MYBs including those involved in regulation of anthocyanin biosynthesis already reported in plants^43^. Strong negative correlation between upregulated expression of candidate repressor MYBs (MYB4-like, MYB34, MYB46, MYB52, MYB61, MYB85 and MYB86) and TAC during S3_G suggests their key role in seasonal induced regulation of anthocyanin accumulation. Moreover, MYB4-like TFs repress anthocyanin biosynthesis either through direct binding of promoter regions of ANS, DFR and UFGT, or displacing the activator MYB in the MBW complex hence reducing the complex to anthocyanin repressor complex^43,45^. Nevertheless, upregulated expression of MYB85 during S3_G may be associated with either activation of repressor MYB4-like TF, or diversion of anthocyanin pathway to lignin biosynthesis at *P*-coumaroyl-CoA branching point, and hence regulating metabolic flux towards anthocyanin biosynthesis^46^. Additionally, novel putative repressors identified here can be further characterized at functional level and explored for genetic improvement of anthocyanin accumulation in tea.

Phytohormones are also important internal factors influencing anthocyanin biosynthesis in plants^47^. ABA regulates anthocyanin biosynthesis via serial activation of PYR/PYL leading to inhibition of PP2C, and triggering of SnRK2 which regulate ABF mediated activation of MBW complex^47^. Interestingly, strong positive correlation between the expression of PYL, PP2C and TFs (MYBs, bHLH), and TAC suggests ABA mediated transcriptional regulation of anthocyanin biosynthesis in tea (Fig. 5A) as earlier reported in blueberry and wolfberry^48,49^. Similarly, significant positive correlation between ABA signaling genes and phenylalanine ammonia lyase also suggest ABA-mediated direct regulation of anthocyanin biosynthesis pathway genes as reported in strawberry^29^. Contrarily, negative correlation between expression of auxin-signaling genes (AUX1, AUX/IAA and SAUR) and activator TFs (MYB, bHLH) during S3_G, affirmed their inhibitory role in anthocyanin biosynthesis^50^. Furthermore, negative correlation recorded between genes involved in ethylene signaling (ETR, EBF1/2, ERF), TAC and day-length, highlighted photoperiod-dependent regulation of anthocyanin accumulation in tea^29^. Our findings corroborate recent study on light-mediated anthocyanin accumulation via induced expression of key regulatory and structural genes involved in anthocyanin biosynthesis^51^.

Moreover, concomitant upregulation of ethylene signaling genes along with repressor transcription factors suggest key role in negative regulation of anthocyanin accumulation in tea^52^. Likewise, negatively correlated expression dynamics of several putative peroxidases (13) and laccases (11) and TAC also suggests their crucial role in seasonal induced anthocyanin degradation in tea^30,53,54^. Interestingly, peroxidase mediated anthocyanin degradation via direct oxidation of anthocyanin molecules, or first deglycosylation of sugar moieties by β-glucosidases followed by peroxidase oxidation (Fig. 5a) has been reported in ornamental kales^21^. Similarly, epicatechin-mediated laccase degradation of anthocyanin via oxidation of epicatechin molecules to quinones followed by polymerization with anthocyanin leading its deglycosylation (Fig. 5a) is known in many fruits^53^. Therefore, positive correlation between upregulated expression of laccases and ANR (responsible for epicatechin biosynthesis) suggest active involvement in anthocyanin degradation during S3_G. Similarly, their upregulation may also be linked to diversion of the substrate (*P*-coumaroyl CoA) to lignin biosynthesis, thus reducing metabolic flux towards anthocyanin biosynthesis^55^. Interestingly, significant homology of six candidate peroxidases with functionally validated peroxidases involved in anthocyanin degradation in brunsfellia, poplar and apple, possibly confirm their significant role in seasonal induced anthocyanin degradation in tea.

## Conclusion

In this study, enhanced purple leaf coloration during S2_GP correlated with higher total anthocyanin content highlighting season’s potential for production of “anthocyanin-rich specialty green tea”. Identification of key DEGs associated with anthocyanin biosynthesis [PAL, 4CL, F3H, DFR and UGT/UFGT], vacuolar trafficking [ABC, MATE and GST] transcriptional regulation [MYB, NAC, bHLH, WRKY and HMG] including novel mechanism of phytohormone signaling genes [PYL and PP2C] and anthocyanin degrading genes [Prx and lac] suggest well-coordinated seasonal controlled metabolism and catabolism in tea. Current findings speculate molecular insights of seasonal induced anthocyanin biosynthesis and degradation in tea (Fig. 4). Wherein, downregulation of key activator TFs, transporters and ABA signaling genes possibly limits the expression of genes involved in anthocyanin biosynthesis reducing total anthocyanin accumulation causing loss of purple leaf coloration in tea. Nevertheless, upregulation of key repressor TFs (MYB4-like, and MYB85), genes involved in hormonal signaling (auxin and ethylene) may inhibit anthocyanin biosynthesis by binding promoter regions of structural genes repressing their activity. Moreover, upregulation of vacuolar peroxidases and laccases possibly degrade anthocyanin by removing glycosyl molecule converting into colorless anthocyanidin causing loss of purple leaf coloration in tea.

Genetic manipulation of key structural and transcription factor genes identified in this study can enhance seasonal independent anthocyanin accumulation and maintain prolong purple leaf coloration. Furthermore, comprehensive genome-wide transcriptional data generated will provide a valuable genomic resource for development of functional molecular markers for expediting breeding and selection of elite anthocyanin-rich tea cultivars.

## Materials and methods

### Plant materials and sampling

In this study, a mature purple tea cultivar (> 15 years old) ‘CSIR-IHBT-PT-17’ grown and maintained under full-sun field conditions at the CSIR-Institute of Himalayan Bioresource technology, Palampur, India (32° 6 52′ N, 76.533° 24′ E) was selected. CSIR-IHBT-PT-17 is a purple-leaf tea cultivar, selected from a natural population of field grown pigmented tea cultivars. Major characteristics of the cultivar include: semi-arbor plant type, purple-green young shoots with two leaves and a bud, which turns completely green as season changes with the exception of purple petiole. The leaves of ‘CSIR-IHBT-PT-17’ are also moderately elliptic with medium serration. Samples (bud with two leaves) were harvested monthly from April to October at 1100HRS-IST (Indian Standard Time), immediately frozen in liquid nitrogen and stored at − 80 °C until analysis. Subsequently meteorological data viz; precipitation, relative humidity, day length and air temperature were recorded during sampling (Supplementary Table S7; Supplementary information).

### Extraction and determination of total anthocyanin content

Total anthocyanin content was determined using a pH differential method as described by Yuan et al^56^ with slight modifications. In brief, frozen shoots (100 mg) were crushed into fine powder in liquid nitrogen, extracted separately in 2 ml each of buffer A (25 mM KCl pH 1.0) and buffer B (400 mM sodium acetate pH 4.5), then centrifuged at 12,000*g* for 15 min at 4 °C. The supernatant was collected and diluted for direct measurement of absorbance at 510 nm and 700 nm. Total anthocyanin content was calculated using the following equation;

$${\text{Total anthocyanin }}({\text{mg g}}^{{ - {1}}} \;{\text{FW}}) \, = ({\text{A }} \times {\text{ MW}}/\varepsilon \times {\text{dilution factor}}$$

where A is the absorbance of the diluted sample calculated as follows;

$${\text{A}}\, = \,({\text{A}}_{{{51}0}} {-}{\text{A}}_{{{7}00}} )_{{{\text{pH1}}.0}} {-}({\text{A}}_{{{51}0}} {-}{\text{A}}_{{{7}00}} )_{{{\text{pH4}}.{5}}}$$

MW is the molecular mass (484.8) of predominant anthocyanin (cyaniding-3-glucoside chloride), while ε is the molar absorptivity (24,825) at 510 nm. Each sample was analyzed in triplicate and the results were expressed as the average of the three measurements.

### RNA extraction, library preparation and sequencing

Based on leaf color transitions and significant variation in TAC, leaf samples harvested in April; S1_RP (Reddish-Purple), August; S2_GP (Dark Gray Purple) and October; S3_G (olive-Green) were used for transcriptome analysis. Total RNA was extracted from each sample using IRIS method^57^. RNA quality and quantity was determined on 1% formaldehyde agarose gel and NanoDrop 2000 spectrophotometer, respectively. Further, RNA integrity (RIN) was determined using an Agilent Bioanalyzer 2100 (Agilent), and RNA samples with A260/A280 ratio > 1.8, A260/A230 ratio > 2.0 and RIN > 7 were considered for library preparation. Equimolar concentration of RNA from three replicates of each sample were pooled for cDNA library preparation using ILLUMINA RNA Sample preparation kit (Illumina Inc., CA, USA) as described by the manufacturer. Libraries were quantified using Qubit 2.0 fluorometer (Invitrogen, USA), while quality assessment was performed using AGILENT 2100 Bioanalyzer (Agilent Technologies, USA). The cDNA libraries were sequenced on ILLUMINA NOVASEQ 6000 (Illumina Inc., CA, USA). Paired-end sequencing format of 100 bp was adopted in this study.

### Transcript assembly and differential gene expression analysis

Raw reads were trimmed to remove adapter sequences, ambiguous nucleotides (N) and low-quality reads (Phred quality score ≤ Q30) using NGS QC Toolkit^58^. High quality clean reads were analyzed according to Tuxedo protocol^59^. In brief, high quality clean reads were assembled using the reference tea genome (*Camellia sinensis* var. *sinensis*^25^ using TOPHAT ver2.1.0. Functional annotation of assembled transcripts was performed by BLAST alignment of the sequences against public databases such as Swiss-Prot, KEGG and TAIR10. GO and KEGG annotations were performed using AgriGO toolkit and KEGG pathway database, respectively^60,61^. The Transcription factor (TF) families were identified using Plant transcription factor database (PTFDB)^62^.

### Identification of differentially expressed genes

Transcripts abundance was estimated in FPKM (**F**ragments **P**er **K**ilobase of transcript per **M**illion mapped reads) and differentially expressed genes with *log*_*2*_ Fold Change (absolute value) ≥ 2 and *p* ≤ 0.05 were considered differentially expressed (DE) using cuffdiff tool in Cufflinks version 2.2.1 with default parameters. GO and KEGG enrichment of differentially expressed genes (DEGs) was performed using publicly available Tea Plant Information Archive (http://tpia.teaplant.org/) database^63^. The corresponding chromosomal locations of DEGs were also determined by mapping them to the chromosomes of *C. sinensis* var. *sinensis* based on their physical distances in the GFF genome files, downloaded from chromosome-scale genome of tea^25^. The physical chromosome maps were visualized using TBtools (a **T**oolkit for **B**iologist integrating various biological data handling tools) VS 1.051 (https://www.tbtools.com) with default parameters^64^.

### Phylogenetic analysis of DEGs involved in anthocyanin degradation

To determine the phylogenetic relationship between DE putative anthocyanin degrading genes and functionally characterized genes from apple, populus and brunsfellia, phylogenetic analysis was performed using MEGA5.2^65^. Sequences were aligned using ClustalW^66^ while phylogenetic tree was constructed using the Neighbor-joining method based on the Tamura-Nei model with bootstrap value kept at 1000. The resulting tree was visualized using iTOL v5 (https://itol.embl.de/)^67^.

### qRT-PCR validation of RNA-seq data

In order to verify the reliability of RNA-seq data, 2 µg of total RNA used in transcriptome sequencing was reversed transcribed using RevertAid H minus First Strand cDNA Synthesis Kit (Thermo Scientific, USA). Gene specific primers were designed with BatchPrimer3 (http://probes.pw.usda.gov/batchprimer3/) and primer sequences are presented in Supplementary Table S8. The qRT–PCR was performed in a StepOnePlus Real-Time PCR system (Applied Bio-system, USA) using SYBR Green Master mix (Applied Biosystem). Considering its consistent expression pattern in a recent study^68^, GAPDH gene was used as internal control. All experiments were performed in three independent biological and technical replicates. Relative expression was calculated using 2^−ΔΔCT^ method^69^.

### Statistical analysis

All statistical analysis was performed using GenStat V15.1 (VSN Intl.).

## Supplementary Information

Supplementary Information

## Data Availability

The nucleotide sequence(s) reported in this article have been submitted to NCBI SRA database under accession numbers; SRR12153423, SRR12153422 and SRR12153421.
