# Supplementary material for: Transcriptional analysis reveals key insights into seasonal induced anthocyanin degradation and leaf color transition in purple tea (Camellia sinensis (L.) O. Kuntze)
Source: Sci Rep. 2021 Jan 13;11:1244. doi: 10.1038/s41598-020-80437-4 (PMC7806957; doi:10.1038/s41598-020-80437-4)
Supplement: Supplementary file 1 — Supplementary Information [file 41598_2020_80437_MOESM1_ESM.zip › Supplementary Materials/Supplementary Information c54bd3a7de98-4ecd-955b-e845a5434af6.docx]

**Supplementary Information: c54bd3a7-de98-4ecd-955b-e845a5434af6**

**Transcriptional analysis reveals key insights into seasonal induced anthocyanin degradation and leaf color transition in purple tea (*Camellia sinensis* (L.) O. Kuntze)**

Tony Kipkoech Maritim^1, 2, 3^, MamtaMasand^1,2^, Romit Seth^2^, Ram Kumar Sharma^1,2^*

^1^Department of Biotechnology, CSIR-Institute of Himalayan Bioresource Technology, P.O. Box No. 6, Palampur (HP), 176061, India

^2^Academy of Scientific and Innovative Research (AcSIR), CSIR-HRDC Campus, Ghaziabad, Uttar Pradesh- 201 002, India

^3^Tea Breeding and Genetic Improvement Division, KALRO-Tea Research Institute, P.O. Box 820-20200, Kericho, Kenya

**Seasonal changes in weather conditions**

Significant seasonal variation in precipitation, relative humidity, temperature, and day-length was recorded between April and October **(Fig. S1A, B)**. July-August recorded the highest average precipitation of ~145.0mm wherein, maximum (130.4mm) was recorded in August, while April-May and September recorded the least at ~15.0mm and 6.2mm respectively. Similarly, relative humidity was high in July-August (~69.5%), followed by September-October at 47.5% and lowest in April/May at 20%. Furthermore, April-June were hotter months with average monthly temperature of ~31.5⁰C, while July/August recorded on average ~30.5⁰C September/October were slightly cooler as ~27.3⁰C Interestingly, June-July recorded up to 13hrs photoperiod while October experienced shorter days of ~10hrs.


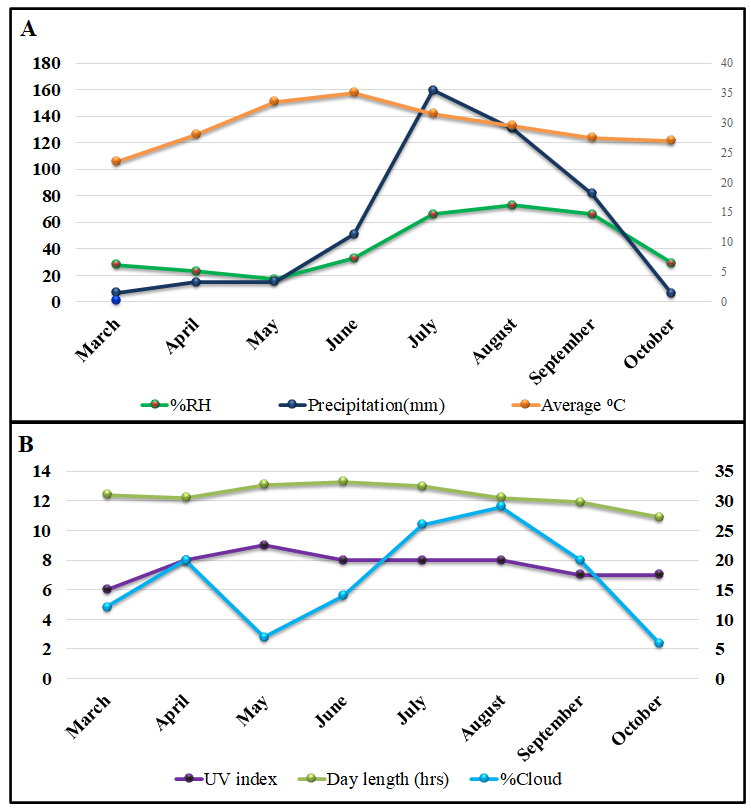


**Supplementary Figure S1**: Average meteorological data collected during sampling (A) Relative humidity, precipitation and temperature; (B) Day length, cloud cover and uv index
